# Nanotechnology in Oral Cavity Carcinoma: Recent Trends and Treatment Opportunities

**DOI:** 10.3390/nano9111546

**Published:** 2019-10-31

**Authors:** Francesca De Felice, Costanza Cavallini, Alberta Barlattani, Mario Tombolini, Orlando Brugnoletti, Vincenzo Tombolini, Antonella Polimeni

**Affiliations:** 1 Department of Radiotherapy, Policlinico Umberto I, “Sapienza” University of Rome, 00161 Rome, Italy; vincenzo.tombolini@uniroma1.it; 2 Department of Oral and Maxillo Facial Sciences, Policlinico Umberto I, “Sapienza” University of Rome, 00161 Rome, Italy; costanza.cavallini@uniroma1.it (C.C.); mario.tombolini@uniroma1.it (M.T.); orlando.brugnoletti@uniroma1.it (O.B.); antonella.polimeni@uniroma1.it (A.P.); 3 Department of Clinical Sciences and Translational Medicine, Tor Vergata University, 00133 Rome, Italy; alberta.barlattani@ptvonline.it

**Keywords:** oral cavity cancer, surgery, radiotherapy, nanotechnology, nanoparticle, imaging, diagnostic exam, photodynamic therapy, sentinel lymph node, research

## Abstract

Oral cavity carcinoma (OCC) remains an ongoing public health problem. Emerging nanotechnology provides alternative treatment approaches. This review covers the up-to-date literature in the human OCC treatment field. We explored the growing body of evidence to reveal novel and highly promising diagnostic and therapeutic applications of nanotechnology in this field. Various types of nanoparticles have been tested for applications in OCC. Imaging modalities in addition to nanocarriers are discussed. The encouraging contribution of lymphotropic nanoparticles contrast in the diagnosis of metastatic cervical lymph nodes needs to be confirmed. The development of the sentinel lymph node procedure and photodynamic therapy may lead to breakthrough therapies in order improve clinical outcomes and quality of life. In this perspective, cancer nanotechnology has the potential to revolutionize the treatment of OCC patients.

## 1. Introduction

Oral cavity carcinoma (OCC) alone accounts for 2.1% of all cancer cases and 1.8% of all cancer deaths among both males and females worldwide [1]. Based on the most recent Surveillance, Epidemiology and End Results (SEER) data, prognosis remains still relatively poor, with a 5-year survival rate of 65% [2]. Surgical resection is the mainstay of treatment [3]. Adjuvant chemoradiotherapy (CRT) or adiuvant radiotherapy (RT) is usually recommended in case of locally advanced disease (pT3-4, pN2-3), positive surgical margins, perineural invasion and lymphovascular invasion [3]. Despite the state of the art includes less invasive surgical techniques, new reconstructive modalities and the intensity modulated RT (IMRT) technique with daily 1.8–2 Gy fractions five fractions per week, combination of these therapies can result in important morbidity [4,5]. To overcome the limitations of standard multimodal approach, over the last years, the rapid development of cancer nanotechnology promises new opportunities in OCC management. This review focuses on current and upcoming options of nanotechnology in OCC patients with special regard to its integration in diagnostic procedures and future treatment strategies. We describe the use of nanotechnology for clinical therapeutic purposes developed to meet the demands for increased survival outcomes and decreased toxicity of treatment. New possibilities in OCC diagnosis and treatment are discussed. The overall purpose is to offer further understanding on the development of nanotechnology in OCC scenario.

## 2. Cancer Nanotechnology

Cancer nanotechnology results from the multidisciplinary field derived from medicine, physics, biology, chemistry and engineering. In its strictest definition (http://www.nano.gov), nanotechnology refers to man-made nanoparticles—with sizes from 1 to 100 nanometers (nm)—potentially useful to treat solid tumors. Detailed physicochemical characterization of nanoparticles is beyond the aim of this review and the reader is referred to the original literature in order to get more technical detail. We only briefly described the main features to understand the scientific basis for the development of nanoparticle-based therapeutics. In essence, the hope is to alter chemotherapeutic agents biodistribution, minimizing localization in healthy tissue and enhancing uptake in malignant cells [6]. Particularly for OCC treatment, nanotechnology-based approaches include nanoparticles, liposomes, hydrogels and liquid crystal as engineered vehicles for drug delivery [7]. They have the potential to penetrate the cell structure and cause better selective accumulation of the therapeutic agent in the tumor tissues. A summary of the main structure, advantages, and limitations of OCC nanocarriers are listed in Table 1 [7,8,9,10,11,12,13,14,15]. 

Globally, these nanomaterials are used due to their biocompatibility, biodegradability, mechanical properties and low toxicity profile. Ideally nanomaterials should be flexible to change their sizes, morphologies and surface properties to enhance tumor accumulation and drugs cell internalization [16]. Typically, nanoparticles cross the cellular membranes by endocytosis via avoiding the drug refluxing back out of the cell—or trough enhanced permeability and retention process using the porous vasculature with leaky endothelium of tumor tissue [16]. However, nanomaterials toxicity still needs to be fully evaluated in humans. There are several limitations concerning both short- and long-term interactions between nanomaterials and biological tissues. Nanomaterials distribution in the body mainly depends on surface characteristics of the particles and the risk of cardio-pulmonary toxicity should be assessed for every newly produced nanoparticle [17]. Although preliminary studies are showing promising results, they have been small and limited to short-term exposure and further analyses are needed to confirm their safety [18].

In general, cisplatin (cis diaminodichloroplatinum, CDDP) is the preferred systemic drug to be administered in combination with RT in fit patients with locally advanced OCC [3]. High-dose CDDP every 3 weeks (100 mg/m^2^ intravenously) with concurrent standard fractionated RT is the most commonly utilized regimen, resulting in survival benefit of approximately 4% compared to other concomitant single agent systemic therapy [19]. CDDP is classified as an alkylating agent and thus, like the alkylating agents, forms intra-strand (95%) or inter-strand (5%) deoxyribonucleic acid (DNA) cross-links [20]. CDDP is a cell cycle non-specific drug most active in the resting phase (phase G0) of the cell. It interferes with DNA repair mechanisms, causing activation of apoptosis pathways [21]. CDDP exhibits poor systemic stability, limited water solubility and frequent side effects (primarily, bone marrow depression, nephrotoxicity and ototoxicity). To overcome these limitations, a ligand-decorated cancer-targeted CDDP-loaded poly(lacticco-glycolic acid)-poly(ethylene)glycol (PLGA-PEG)/NR7 nanoparticles have been formulated [22]. Preclinical studies proved its capacity to facilitate drug accumulation in OCC tissues and assure a rapid intracellular uptake. Human exploration of the application of PLGA-PEG/NR7 in routinely clinical application is needed and further research work is fundamental.

The utilization of nanoparticles represents also a plausible resource to intensify (C)RT treatment. From a radiobiological point of view, prolonging tumor exposure to drug improves (i) synergistic actions of chemotherapy and ionizing radiation, (ii) inhibition of intra-fraction tumor growth and (iii) promotion of normal tissues repopulation [23]. This strategy could directly ameliorate local control rates limiting normal tissue exposure and thus maximize the therapeutic ratio.

## 3. Diagnostic Procedures

At present, the combination of information collected from radiologic imaging with clinical examination allows for the most accurate stage disease at diagnosis [3]. Magnetic resonance imaging (MRI) with intravenous contrast of primary and neck and contrast-enhanced computed tomography (CT) of chest with or without primary and neck evaluation are part of the routine pre-treatment multidisciplinary work-up. 18F-labeled fluoro-2-deoxyglucose (18F-FDG) positron emission tomography (PET)-CT should be considered in advanced stage or in case of an uncertain staging. 

Over the years, with the advancement in nanotechnology, different types of nanoparticles have been investigated as specific contrast agents in diagnostic exams to improve their accuracy, sensitivity and specificity. We focused on novel contrast agents for OCC diagnosis.

### Lymphotropic Nanoparticles

Surely an accurate nodal staging has a predominant role on the treatment strategy decisions and neck dissection is strictly depended on detection of suspicious/certain metastatic node(s) at diagnosis. Traditionally, according to the nomenclature of the American Academy’s Committee for Head and Neck Surgery and Oncology, cervical lymph nodes are divided in six levels, including submental and submandibular group (level I), upper jugular group (level II), middle jugular group (level III), lower jugular group (level IV), the posterior triangle group (level V) and the anterior compartment group (level VI) [24]. Despite diagnostic recommendations for differentiating between benign and pathological lymph nodes include enhancement pattern, shape and dimensions, such as maximum transverse diameter and/or ratio between the maximum longitudinal and the maximum transverse diameters, standard MRI contrast imaging is prone to report relatively high false-negative rates (15–25%) in the cervical lymph nodes characterization [25]. A spherical lymph node larger than 10 mm in its minimum transverse diameter with central necrosis and/or extracapsular spread is an indicator for lymph node metastasis. But one of the most challenging questions is whether a small (5–10 mm) lymph node without necrosis or extracapsular spread should be or not should be considered malignant [26]. This issue is a fertile ground for research.

Lymphotropic nanoparticles are a relatively new class of MRI contrast agents that are likely to improve diagnostic exam sensitivity [18]. Ferumoxtran-10 (Combidex; Advanced Magnetics Inc., Cambridge, MA, USA; Sinerem/Combidex; Radboud University Nijmegen Medical Center, Nijmegen, The Netherlands) is a biodegradable ultrasmall superparamagnetic iron oxide (USPIO) particle covered with low-molecular-weight dextran with a diameter of 17–21 nm. It should be administered intravenously slowly over a period of 30 min, mainly to avoid hypotensive reactions. Ferumoxtran-10 has a long blood half-life (up to 30 h) and enters the physiological iron metabolic pathway through transferrin, ferritin, hemosiderin, and hemoglobin [27]. In the late phase of its distribution, it can be used to evaluate lymph nodes [28]. Due to its small size, ferumoxtran-10 can easily cross the capillary wall and, once in the interstitium, it is cleared by draining lymphatic vessels and localizes within lymph nodes, allowing robust characterization of nodes independent of the size criterion [29]. Normal-sized metastatic nodes can be differentiated from reactive nodes, based on the same signal intensity between pre- and post-contrast MRI images [29]. In fact, in metastatic nodes, macrophages are partially or completely replaced by malignant cells and therefore the physiological phagocytotic process, responsible for signal reduction on post-contrast images in normal nodes, is not maintained [29]. Patterns of node signal-intensity changes—from a benign lymph node with homogenous uptake and signal drop to a completely replaced pathologic node without contrast uptake—have been nicely described and tabulated [27]. Details are shown in Table 2. 

In this context, normal-sized lymph node (less than 1 cm), that represents ordinarily a potential cause of false-negative result in case of size criteria alone, can be detected metastatic. Data from interpretation of the MR images of 147 patients have recently demonstrated that ferumoxtran-10 yielded an overall accuracy of 93%, sensitivity of 96% and specificity of 87% in diagnosis of pathologic or negative nodes [27]. In this open-label multicenter study, MR imaging protocol included the acquisition of transverse T1-weighted spin-echo, transverse T2-weighted fast spin-echo and transverse T2*-weighted gradient-echo images. Post-contrast MR images were obtained 24–36 h after the ferumoxtran-10 administration. No severe adverse events occurred in this series. Interestingly, 29 patients of the 147 patients included in the study had head and neck cancer and ferumoxtran-10 performed best in this setting of cases, highlighting its potential role in the characterization of nodal status in OCC management [27]. 

Several minor series reported similar positive results on ferumoxtran-10 utility in characterizing head and neck adenopathy [25,30,31,32]. For instance, in a cohort of head and neck cancer patients from the University of Iowa, MRI with ferumoxtran-10 of the head and neck region and subsequent neck dissection or fine-needle aspiration biopsy were performed [30]. All patients underwent MRI within 14 days before and 24–36 h after ferumoxtran-10 administration. Lymph nodes were defined metastatic when persistent hyperintense signals on both pre-contrast and post-contrast heavily T2*-weighted gradient echo imaging was recorded. MR images were then correlated with histological findings. Pathological staging of adenopathy was used as the true staging. Histological correlation was possible for a total of 101 lymph nodes identified by MRI exam. In total, a sensitivity of 95% and a specificity of 99% were achieved. MRI with ferumoxtran-10 assessed correctly 99 nodes. Only one false-positive result and one false-negative result occurred [30]. 

Mack et al. [25] reinforced data from previous study. They demonstrated an important USPIO-enhanced MRI effect in the management of head and neck cancer. Thirty consecutive patients (n = 5 with OCC) were included. Ferumoxtran-10 enhanced MRI was performed before and within 24–36 h after administration of the USPIO contrast agent. Results showed a correct diagnosis in 96.3% in the level-by-level MRI/histopathologic exam correlation.

Similarly, in Curvo-semedo et al. [32] cohort of 20 patients with head and neck cancer, ferumoxtran-10-enhanced MRI resulted useful in nodal staging. Authors performed a direct comparison between MRI and pathological examination, evaluating the diagnostic value of USPIO particles. Patients underwent MRI before and 24–36 h after intravenous infusion of ferumoxtran-10. Final analysis showed an accuracy value of 73%, a sensitivity value of 100%, but a low specificity (55.3%). No false-negative cases were recorded. However, it should be stressed that the selection of the lymph nodes to be correlated was based solely on MRI examination. During surgical procedure, MR images were available in the surgical suite to properly map the MRI metastatic nodes, removed them and then separately sent them for pathologic examination.

Overall, these results are in agreement with a multi-institutional phase III clinical trial, that was designed to establish properly the correlation between MRI and histology in patients with proven head and neck squamous cell carcinoma [31]. To note, the primary site was oral cavity in 20 out of 81 cases. All patients underwent MR examination before and 24–36 h after contrast administration and had neck dissection within 15 days of post-contrast MR study. On post-contrast MRI, a lymph node was considered pathologic if it showed high signal intensity on T2*-weighted images, whatever its size. Correlation analysis between imaging and pathology was performed in 129 lymph nodes. USPIO-enhanced MRI sensitivity and specificity were 95% and 86%, respectively. Only 4 false-negative cases were found and this event was attributed to the presence of micro-metastasis (inferior to 3 mm).

Briefly, ferumoxtran-10 MRI seems to be more accurate in detecting minimal metastatic nodal disease particularly in normal sized lymph nodes. For sure the additional time needed to perform the post-contrast MRI—due to the maximum iron peak in lymph nodes 1–2 days after injection—represents the main impediment to more liberal use of this contrast imaging.

## 4. Treatment Opportunities

**Figure 1:**
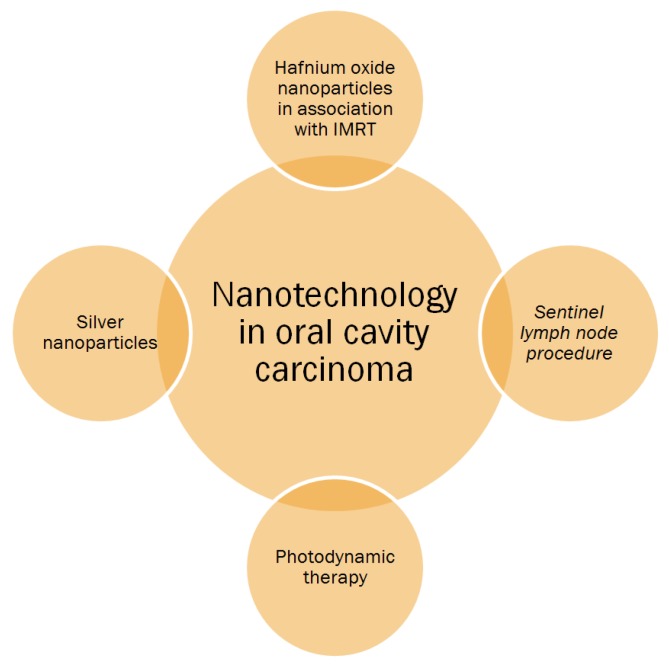
Nanotechnology applications in oral cavity carcinoma.

Several opportunities to create safer and more effective therapeutic modalities are under evaluation in human. This section has been divided into three categories, which include viable alternative cancer therapies in OCC. Figure 1 summarizes these nanotechnology applications.

### 4.1. Ongoing Clinical Trials

There are a huge amount of in vitro and in vivo efficacy and safety data in OCC models, but they have not been translated into clinical use. Very few current and proposed trials are starting to incorporate nanoparticle formulations into OCC management. 

A phase I study (NCT01946867) has been designed to test the safety and tolerability of NBTXR3, a crystalline solution of hafnium oxide nanoparticles, in association with IMRT in elderly OCC patients unfit for standard CDDP-based chemotherapy [33]. NBTXR3 enters tumor cells and yields an increased cell-localized energy deposit upon RT exposure. 

Patients receive a single intra-arterial or intra-tumor injection of NBTXR3 on day 1 followed by IMRT starting 24 h later (on day 2), up to total dose of 70 Gy over 7 weeks (2 Gy per single fraction). The hope is to increase tumor cell killing and complete OCC shrinkage allowing a definitive treatment and preservation of surrounding tissues. Evaluation of the objective response rate and the complete response rate by MRI using response evaluation criteria in solid tumors (RECIST) and the tumor volume estimation (length × width × depth) is planned. Local progression free survival (LPFS) and progression free survival (PFS) will be estimated. Those patients whose tumor has not shrunk more than 50% of the baseline tumor volume, will stop IMRT and may have a salvage surgery to primary lesion. Exclusion criteria include, but not limit to, tumor-related dyspnea, tumor ulceration which implies vascular risk and concurrent treatment with any other anticancer therapy (chemotherapy, immunotherapy, targeted therapy, gene therapy). Preliminary results were presented during 2019 ASCO annual meeting. Primary endpoints of phase I were determination of recommended phase II dose and dose limiting toxicities (DLT). Data showed that NBTXR3 activated by RT is safe and well tolerated, reflecting a promising future treatment. Dose-escalation was completed and no DLT as well as severe adverse events were recorded. In total, adverse events occurred in 5 cases, including one patient with NBTXR3-related grade 1 asthenia, and four patients with intra-tumor injection-related sequelae (n = 1 grade 2 oral pain; n = 1 grade 1 tumor hemorrhage; n = 1 grade 1 asthenia; n = 1 grade 1 injection site hemorrhage). Interestingly, among the 13 evaluable patients treated at NBTXR3 doses ≥ 10%, 9 patients achieved a complete response of the injected lesion. To confirm this encouraging efficacy phase II study has started [33]. 

Recently, researchers have explored the role of silver-based nanoparticles in the therapeutic window of cancer treatment. Several reports suggested that silver-based nanoparticles were able to inhibit the proliferation of different cancer cell lines, including colon, breast, liver, lung and glioblastoma [34,35]. Thus, probably, there is a preclinical rationale behind silver-based nanoparticles potential anticancer effect, but actually the detailed biochemical mechanism of this anticancer activity is still unknown. Two main mechanisms are described, suggesting a multiple pathways anticancer activity: (i) it seems that silver nanoparticles directly induce both DNA damage in a dose-dependent manner and cell cycle arrest in S phase (synthesizing DNA); (ii) on the other hand, silver nanoparticles may interfere with replication process, resulting in collapsed replication forks which can lead to cell death [34]. Recent studies have also assessed the effects of silver nanoparticles in combination with ionizing radiation [35]. Promising preclinical results already have been achieved with silver-based nanoparticles used as radiation sensitizers for cancer therapy in breast cell lines [35]. Compared to single treatment, the combination of silver nanoparticles and RT was more effective for tumor growth inhibition, inducing DNA and oxidative damage in malignant cells while preserving non-cancerous cells [35]. Based on these data, a synergistic effect could be also hypothesized in humans but more detailed research is needed to define the exact mechanism of action and the potential oncology applications in daily clinical practice. The ability to control the unique toxicity profile of silver nanoparticles is an important issue. Exposure to silver-based nanoparticles causes dose-dependent toxicities, mainly linked to oxidative stress leading to mitochondria-dependent apoptosis [36]. However, despite there is a lack of ongoing and completed clinical trials in neoplastic patients, silver-based nanoparticles seem to be an effective treatment. Firstly, tested in a patient with highly refractory metastatic head and neck squamous cell carcinoma, silver nanoparticles showed high efficacy leading to complete resolution of primary lesion and metastasis to the liver and lung, persisting for 18 months [37]. Actually, this was not a scientific report. Once metastatic disease was diagnosed, the patient started to manufacture and consume a silver-based nanoparticles solution daily for 3 months. During this time period, the patient felt better—his performance status improved from 3 to 0—and re-gained his functional capacity. He underwent restaging diagnostic exams and there was no radiological evidence of disease. Blood parameters, as well as renal function and liver function were also assessed and resulted normal. Surely, this case report is not conclusive. Further patients should be treated to validate silver nanoparticles safety and efficacy. Taken together, these results appear very promising, but are hypothesis generating rather than confirmatory. Efforts should be made to evaluate the real effect of nanoparticles and their interaction with standard treatment modalities on OCC patient survival.

### 4.2. Sentinel Lymph Node Analysis 

Future perspectives might also focus on the development of nanotechnology approaches for sentinel lymph node analysis. 

This lymph node localization procedure is mainly described and routinely used in other malignant disease, including melanoma and breast cancer [3]. Traditional procedure consists in the peritumoral injection of a radiotracer to properly map the first lymph node—referred to as the sentinel node—that receives drainage from primary tumor and subsequently the remaining lymph node basin. If the sentinel node results negative on histological examination, there is a significant low incidence risk of metastases to the rest of the lymphatic bed and prophylactic lymphadenectomy can be avoided [38]. Surely a substantial experience is required to achieve a high success rate with this technique [39]. It has been reported a strong relationship between the number of procedures performed by surgeon and the success in identifying sentinel node. The surgeon who performed the most procedures achieved a higher success rate compared to the surgeon with initial experience in intra-operative lymphatic mapping (96% versus 72%; p < 0.01) [39].

At present, sentinel lymph node biopsy represents an alternative to elective neck dissection for identifying occult cervical metastasis in patients with early (cT1-2) OCC [3]. Such patients with early (cT1-2) OCC present a common therapeutic dilemma in the management of the regional nodes. As it is reported in literature, the overall rate of occult lymph node metastasis is 20–30% in early stage OSCC patients. Therefore 70–80% of patients with cN0 neck will not benefit from elective neck dissection [40]. However, it remains unclear whether sentinel lymph node analysis can definitely replace elective neck dissection in treatment of OCC patients with clinically N0 neck. In a recent meta-analysis of 66 studies (n = 56 prospective trials, n = 10 retrospective analysis) comprising 3566 patients, authors showed that the sentinel lymph node procedure yielded a pooled sensitivity of 0.87 (95%CI: 0.85–0.89; I^2^ = 20.5%) and a pooled negative predictive value of 0.94 (95% CI: 0.93–0.95; I^2^ = 0) in early stage OCC [33]. Sentinel lymph node histopathologic evaluation was performed according to the gold standard reference for sentinel lymph node metastasis diagnosis (hematoxylin and eosin staining, immunohistochemistry, serial sectioning), in all included studies [41]. Studies were of moderately high quality. Selection bias was high and mainly related to retrospective analysis and absence of randomization in prospective studies. Authors conducted a subgroup analysis to better define the single study effect on the diagnostic efficacy of sentinel lymph node procedure [41]. The following variables were investigated: average of sentinel lymph nodes harvested (low < 2 versus medium ≤ 2 and < 3 versus high ≥ 3), sentinel lymph node pathology methods (immunohistochemistry versus not immunohistochemistry; serial sectioning versus not serial sectioning), type of reference test (neck dissection versus follow-up), sentinel lymph node tracer (single tracer versus multiple tracers), study design (prospective versus retrospective) and publication year (early 2000–2008 versus late 2009–2016). Immunohistochemistry (0.88, 95%CI 0.86–0.90 versus 0.77, 95% CI 0.68–0.85), neck dissection (0.90, 95%CI 0.87–0.93 versus 0.85, 95%CI 0.82–0.88) and early publication (0.92, 95%CI 0.87–0.95 versus 0.86, 95%CI 0.83–0.88) subgroups were significantly more sensitive compared to their counterpart [41]. Based on these results, authors concluded that sentinel lymph node biopsy with immunohistochemistry procedure represents a valid alternative to elective neck dissection [41]. 

Nowadays it is accepted that sentinel lymph node status can drive, in those centers where expertise for this procedure is available, surgical treatment planning in early OCC: patients with a positive sentinel lymph node must undergo a completion neck dissection, while those without should follow a close observation [3]. 

Obviously the watchful waiting approach is not associated with surgical complications—mainly shoulder syndrome (winged scapula, shoulder pain, and limited arm abduction)—resulting from elective neck dissection [42,43,44,45]. Despite preservation of the C2-C4 cervical plexus integrity, approximately 30% of patients undergoing elective neck dissection referred the shoulder syndrome [43]. To better rate and standardize the shoulder function quality the Constant-Murley score [45] is used. It is a 100-point scale composed of a number of individual parameters, including pain (15 of the total 100 points), activities of daily living in term of work, recreation, sleep and position (20 of the total 100 points), range of motion testing abduction, flexion, internal rotation and external rotation (40 of the total 100 points) and shoulder power (25 of the total 100 points) [45]. The higher the score, the better the quality of the shoulder function. Several series compared secondary morbidity between sentinel node biopsy and elective neck dissection in clinically negative neck. Hernando et al. [42] prospectively enrolled 73 OCC patients with stage I-II squamous cell carcinoma. They compared 29 sentinel node biopsy patients with 41 elective neck dissection patients. Murer et al. [43] analyzed two groups consisting of 33 patients after sentinel node biopsy and 29 after elective neck dissection. Schiefke et al. [44] evaluated the functional status in 24 patients after sentinel node biopsy and 25 patients after elective neck dissection. Overall, their conclusions were that functional outcomes assessed by Constant-Murley score after sentinel node biopsy were significantly better than after elective neck dissection.

**Figure 2:**
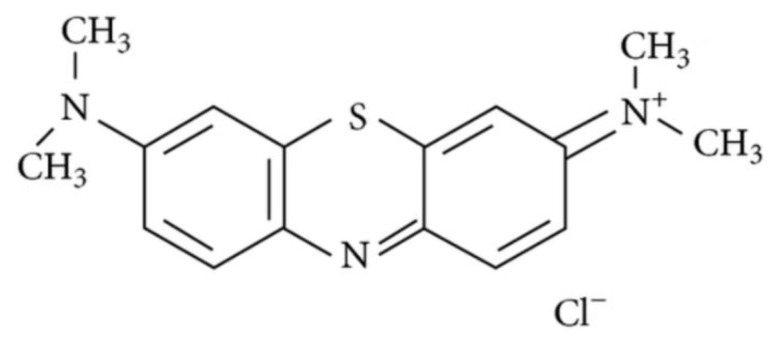
Chemical structure of methylene blue dye.

Usually, sentinel lymph node analysis adopts a preoperative lymphoscintigraphy with radio-labelled particles combined with intraoperative gamma probe localization and an injection of blue dye [42,43,44,45]. On the day of surgery, the patient had a minimum of two peritumoural injections of radiotracer, depending on the location and size of the tumor. The identification of radioactive colloid accumulation within sentinel lymph node using a gamma ray counter supports neck node dissection in patients with nodal negative early OCC [46,47]. Interestingly, a detection of sentinel lymph node using methylene blue dye alone has been proposed in developing countries due to their limited availability of lymphoscintigraphic facilities [48]. Methylene blue dye is effective, low cost, easily available and with a good safety profile [49] (Figure 2). 

The largest study of sentinel node biopsy in early OCC included 94 patients (75 men and 19 women, with a median age of 45 years) with cT1-2 cN0 disease [48]. This prospective study was conducted in a large tertiary care cancer centre in India. After peritumoural injection of blue dye (methylene blue 1–2 mL) and resection of the blue node, selective neck node dissection was performed in all patients. Primary endpoint was to estimate the concordance between the sentinel node and the elective neck dissection specimens concerning metastatic nodal involvement [40]. Lymph nodes metastasis were described in level IB (48.6%), IIA (37.1%), III (8.6%) and IA (5.7%). Results showed a good sensitivity (84.6%) and negative predictive value (93.9%) of the procedure and defined immunohistochemistry as the preferable technique to detect micrometastasis within lymph nodes [48]. The main limitation is the blue dye spillage in tissue planes, even though one-millimeter injection seems to reduce spillage while still maintaining an oncologic adequate result. Regardless, sentinel lymph node biopsy with blue dye and radioactive tracers involves the use of ionizing radiation, is invasive and requires surgical skill.

In this scenario, nanotechnology has the potential to enable sentinel lymph node procedure without the use of radioactive agent and without compromise the good overview of the working surgical field [50]. 

**Figure 3:**
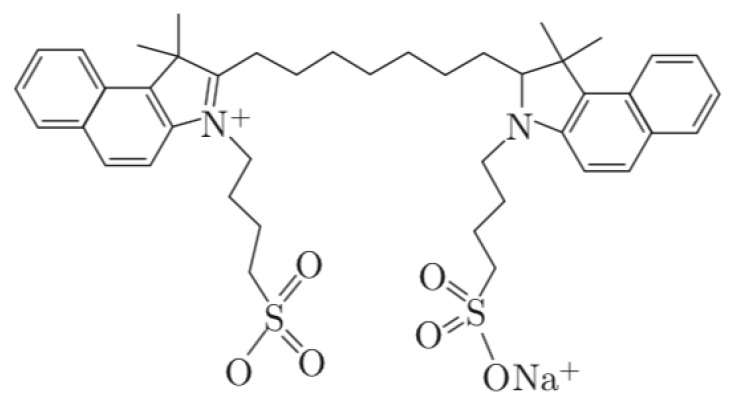
Chemical structure of indocyanine green [52].

Indocyanine green (ICG, CAS number: 3599-32-4) is an amphiphilic tricarbocyanine dye with a molecular weight of 774.974 g/mol [51]. Its basic chemical structure is depicted in Figure 3. 

Indocyanine green has been approved by the Food and Drug Administration (FDA) in late 50s and is routinely used in humans to determine cardiac output, measure hepatic function and study retinal vessels [52]. After injection, indocyanine green is quickly eliminated by liver and bile duct. It is well tolerated and has an excellent safety profile. Serious events include rare anaphylaxis. Indocyanine green has photophysical properties and emits fluorescent light peaking at about 800 nm, making it highly appropriate for imaging applications. This characteristic fluorescence spectra within a near-infrared optic window is utilized in fluorescence imaging. To overcome its short half-life (2–4 min), poor hydrolytic stability, concentration-dependent aggregation, lack of target specificity and poor photo-stability, indocyanine green can be incorporated into different nanoparticle structures, including magnetic, lipid-based, polymer-based nanoparticles [52]. Near-infrared fluorescence (NIRF) imaging is an attractive modality for sentinel lymph node mapping, mainly due to its ability to bypass the blue dye’s radioactive issues, low spatial resolution and allergic reactions. Therefore, over the years, indocyanine green-loaded small nanoparticles (inferior to 50 nm in diameter) have been tested as a surrogate for radioactive colloid and blue dye imaging to map sentinel lymph node. NIRF using the fluorescent dye indocyanine green has been successfully applied in melanoma, breast cancer and gynecological cancers, including cervical cancer and vulvar cancer [53,54,55]. Preclinical work suggested that liposome-encapsulated indocyanine green, both glucosylated liposome-encapsulated indocyanine green and mannosylated liposome-encapsulated indocyanine green, have improved stability and fluorescence signal compared to “free” indocyanine green [56]. These tracers were synthesized with the same molar ratio composition, but preparation of modified liposome was performed by incorporation with glucosylation and mannosylation using p-aminophenyl-β-D-glucopyranoside as glucose and p-aminophenyl-α-D-mannopyranoside as mannose [56]. 

The use of indocyanine green in the diagnosis of sentinel lymph node has also been described in head and neck region. Bredell et al. evaluated the potential application of indocyanin green in 8 patients with OCC and clinical N0 status [57]. Location of primary tumor was the retromolar trigonum in 1 patient, tongue in 2 patients, maxilla in 3 patients and floor of mouth region in 2 patients. In all cases, surgical resection of the primary tumor and unilateral elective neck dissection were planned. Indocyanin green (1 mL per patient with at least five injection points) was injected around the tumor lesion. After 3–5 min, an infra-red video camera was directed to the cervical area to identify the sentinel lymph node. Neck dissection was performed, once sentinel node was marked and sent separately for pathologic examination [57]. Following this procedure, sentinel lymph node was identified in all patients. Fluorescent tissue was identifiable when 5 mm or less tissue covered the node. Authors reported tracer uptake in the submandibular gland, but it was easily distinguished as non-lymphatic tissue.

Another single-institution clinical trial demonstrated that NIRF imaging can successfully be used to detect draining lymph nodes in head and neck cancer patients [58]. A total of 10 consecutive patients with oral cavity (n = 8, including tongue in 7 cases and retromolar trigone in 1 case) or oropharyngeal (n = 2 tonsil) cancer and a clinical and radiological negative node of the neck were eligible for participation in the study. To increase fluorescence intensity and hydrodynamic diameter, the complex indocyanine green adsorbed to human serum albumin was used to map sentinel lymph node [59]. NIRF imaging was performed with a miniaturized version of the fluorescence-assisted resection and exploration (Mini-FLARE) imaging system. Mini-FLARE is a general-purpose optical imaging platform that is provided with two wavelength-independent light sources (centered at 600 nm and 760 nm). This system permitted the surgeon to distinguish otherwise invisible structures within the surgical field [58]. After exposure of the neck, the indocyanine green adsorbed to human serum albumin (1.6 mL) was injected around the tumor at four quadrants. There were no adverse reactions. During the neck dissection, fluorescence of node levels I–IV was measured the Mini-FLARE imaging system. Routine histopathologic analysis (hematoxylin and eosin staining) of sentinel lymph nodes was performed. In total 3 patients had positive lymph nodes. All but one was mapped as sentinel node. One case was a false-negative: the metastatic lymph node was not the first-tier node and had not a fluorescent signal [58]. Authors provided potential explanations for this false-negatively staged patient. Firstly, they ascribed the false-negative results to a time issue: probably in case of multiple drainage patterns, sentinel lymph node become fluorescent later and maybe NIRF imaging should be performed later. Furthermore, the 5 mm depth sensitivity of the system will probably hamper fluorescent node identification. Evidence of skip metastasis should also be contemplated. Lastly, authors possibly related false-negative rate to procedure learning curve [58]. 

Another approach recently presented by van der Poel et al. is based on an imaging agent that is both radioactive and fluorescent [60]. This hybrid multimodal radiocolloid—indocyanine green-^99m^Tc-NanoColl—was developed to better monitor the pharmacokinetics and bio-distribution of the fluorescent agent. The radioactive component is used preoperatively to localize sentinel lymph node and intraoperatively to guide surgical procedure. The fluorescent component helps to better identify the exact location of the sentinel lymph node during surgery [60]. 

In summary, there are different nanoparticle structures useful to incorporate indocyanine green in order to improve accumulation in sentinel lymph node, protract tracer circulation and improved its stability to facilitate NIRF imaging. Prospective studies are needed to assess their reliability and true applicability in OCC.

### 4.3. Photodynamic Therapy 

**Figure 4:**
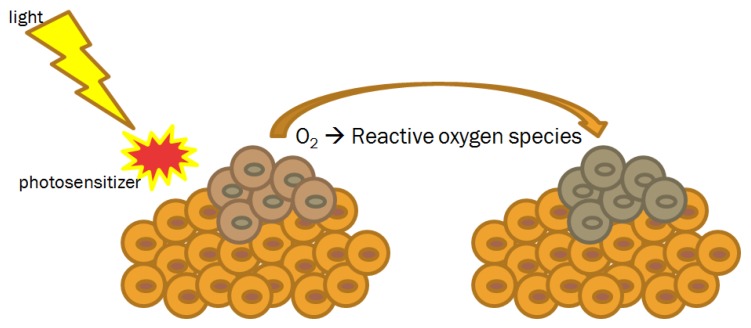
Photodynamic therapy: mechanism of action.

First introduced more than one hundred years ago, photodynamic therapy (PDT) is mainly used in dermatologic disease, including inflammatory, infectious and neoplastic clinical conditions [61,62,63,64,65,66]. To induce its cytotoxic effect, PDT requires the simultaneous presence of three components, including the photosensitizer, the light and the oxygen (Figure 4).

Traditionally, in the case of oxygen levels inferior to 2%, cells result resistant to PDT effect [65]. In general, two main photo-oxidative reactions are described in literature [67]. Type I reactions produce highly reactive radical cation and neutral radicals that can directly interact with substrate molecules, such as DNA bases. In type II reactions, the formation of photo-excited states activates molecular oxygen by electron or energy transfer reactions [68]. The subsequent consequence is the production of reactive oxygen species that undergo a rapid cascade of chemical changes causing damage at cellular level and then on tissue.

Theoretically, every light source with proper spectral characteristics can be adopted in PDT. For instance, laser dyes Rhodamine B (RhB) and sulforhodamine B (Kiton Red S; KRS) are frequently used. These light sources generate up to 7 watt of red light. The amount of energy delivered depends on two parameters: the duration of delivery and the dose rate of light. The greater dose rate, the greater PDT cytotoxicity effect appears.

The ideal PDT photosensitizer should demonstrate (i) a selective retention with a high tumor-to-tissue ratios (2:1, 3:1, 4:1, 5:1); (ii) high quantum yields of singlet oxygen; (iii) adequate penetration depth in tissue; (iv) photolability, in order to damage tumor tissue while minimizing damage to surrounding normal tissues [65]. Photosensitizer therapeutic index is mainly related to its lipophilicity and hydrophilicity and can be improved by coupling the sensitizer to nanoparticles. The vast majority of photosensitizers have a heterocyclic ring structure similar to that of hemoglobin [61]. They can be administered systemically, topically or injected locally. Because of a more stable and defined mixture, at present, the most commonly used photosensitizers are porfimer sodium (photofrin II), meta-tetrahydroxyphenylchlorine (m-THPC, Foscan) and 5-aminolaevulinic acid (5-ALA).

The potential application of PDT in the armamentarium of head and neck cancer treatment was soon realized [69]. A recent systematic review searched relevant clinical studies on PDT efficacy in treatment of head and neck cancers from 1985 until 2015 [69]. In total 12 studies representing 465 patients were included. Photofrin (n = 6 studies), 5-ALA (n = 1 study), hematoporphyrin derivatives (n = 3 studies) and m-THPC (n = 2 studies) were used as photosensitizers. Photosensitizers were administered in intravenous form in all studies but one in which 5-ALA was applied topically. The vast majority of studies (n = 9) used pumped dye as laser source. The remainder used light-emitting diode (n = 1 study), diode (n = 1 study) and gold vapor laser (n = 1 study). Malignant lesions were illuminated with a median total light dose of 50 J/cm^2^ (range 20–100 J/cm^2^). Wavelengths ranged from 628 nm to 652 nm and power density from 80 mW/cm^2^ to 500 mW/cm^2^ [69]. PDT technique was simple, relatively fast (treatment time depended on photosensitizers ranging from one minute up to 143 min) and was safely performed in outpatient clinic. After PDT, complete response was achieved in most cases (> 75% in 9 studies) [69]. The most significant side effect was skin photosensitivity, which lasted up to 6 weeks. To note, several other acute complications were described, such as local pain, burn wounds, trismus, swelling, erythema, edema, pruritus, nausea and vomiting [69].

Malignant lesions of the oral cavity are attractive for PDT, principally due to PDT advantage to not overlap toxicities with standard therapy or itself. Over the years, there have been different publications describing PDT in OCC treatment [70-74]. Details of the main clinical experiences are listed in Table 3. 

Most data were retrospectively acquired. Globally, PDT was extremely effective. The vast majority of studies reported high and durable complete response rate, as well as an excellent cosmetic and functional outcome. Interestingly, primary oral tongue lesions reacted better than other oral cavity sub-sites to PDT, probably due to the relative tissue homogeneity and the relative illumination ease of use. Moreover, success rate seems to be related to early T stage and superficial lesion (depth of invasion < 5 mm). 

Hopper et al. study is the only article that reports success rates for sub-sites [74]. Authors conducted an open-label multicentre study to test the efficacy and safety of mTHPC-mediated PDT in patients with histologically proven primary squamous cell OCC [74]. Early stage T1-2 N0 lesions, without evidence of distant metastasis, were included. Photosensitizer (0.15 mg/kg body weight) was administered by slow intravenous injection. Four days after injection, tumor was illuminated using a laser light of 652 nm wavelength at a fluence rate of 100 mW/cm^2^. Primary end point was tumor response after treatment. Complete response was defined as the disappearance of disease in treatment site. One hundred twenty-one patients received PDT and 114 were included in the final efficacy analysis. Overall, a complete response was achieved in 85% of patients and it was maintained in 85% of responders at 1 year and in 77% at 2 years. Complete response rates were higher for anterior tongue, floor of mouth, lip and buccal mucosa tumors, accounting 93%, 89% 83% and 83%, respectively. One- and 2-year survival rates were 89% and 75%, respectively [74]. Mild to moderate pain was recorded in 82% of cases. Pain was localized at treatment site and persisted for a median period of 3 weeks. Skin photosensitivity reactions were reported in 13% of patients.

Recent PDT advancements include the interstitial technique for the treatment of patients with deep lesions [75]. With interstitial PDT, light fibers are inserted directly into the tumor lesion, maximizing light dose to target volume and sparing adjacent healthy tissues. 

Several research groups have confirmed interstitial PDT effectiveness in the treatment of oropharyngeal cancers [75]. However, to our knowledge, there are still no data regarding interstitial PDT in OCC.

In summary, despite available evidence in literature supports PDT as safe option for OCC treatment, at present there is a lack of consensus regarding its routinely clinical indication.

## 5. Conclusions

Since the development of nanotechnology decades ago, considerable progresses have been made in several important oncologic aspects, including OCC management. Considering that active research in diagnostic exams and technical advancement, in both surgical and radiation techniques, in nanotechnology field is growing every year, we performed a synthesis of the available clinical data and a basis for further considerations.

This narrative review suggests the potential role of nanoparticles in patients with OCC. MRI contrast lymphotropic nanoparticles has the potential to be a sensitive and specific method to better discriminate minimal metastatic nodal disease in normal sized lymph nodes. Fluorescence imaging application of indocyanine green seems to be safe, simple and useful. Sentinel node biopsy in OCC patients with clinically staged N0 appears to be related to less morbidity and similar prognosis rates compared to elective neck dissection. Due to their pre-clinically proven impact on the five “Rs” in radiobiology (radiosensitivity, repair, reoxygenation, redistribution and repopulation), the utilization of nanoparticles as radiosensitizers in association with IMRT has shown great promise in clinical research. Photodynamic therapy seems ideally suited especially to recurrent disease with minimal related-toxicity. It is important to underline several limitations including the low number of clinical articles on that topic and methodological issues. Further investigation is warranted to confirm these results using methodologically robust designs.

In summary, there is great hope that nanotechnology can offer an array of new diagnosis and potential translational therapeutic opportunities over conventional approaches to advance clinical indications and treatment efficacy in OCC. 

## 6. Direction for Future Research

Nanotechnology can be effectively applied in OCC treatment. Exploitation of cancer nanotechnology has large potential for improving clinical outcomes and quality of life in OCC patients. Continued interdisciplinary collaborative research involving medical specialists (oral and maxillofacial surgeons, radiation oncologist, clinical oncologists, radiologist, pathologist, dentistry, rehabilitation physician, audiologist, and pain specialist), healthcare professionals (physiotherapist, speech and language therapist, dietitian, clinical nurse specialist, lymphoedema nurse specialist), biomedical engineers and cancer biologists is essential to concretely realize the benefit of OCC patients and effectively improve their care and satisfaction. 

**Figure 5:**
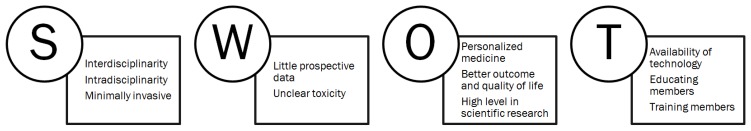
Swot analysis of nanotechnology in the treatment of oral cavity carcinoma.

Figure 5 summarizes strengths, weaknesses, opportunities and threats—swot analysis—of nanotechnology in OCC treatment, based upon the published literature discussed above. 

It is a suggestion to help both clinicians and researchers to develop a full awareness of all the factors involved in this innovative approach and guide appropriate clinical trials design. Improving clinician’s knowledge about the concrete effectiveness of nanotechnology in OCC scenario should represent a major goal. In order to offer unbiased data for shared decision making and not generate false impression, this goal should be reached by high-quality and evidence-based research. It is essential to establish which population should be included. Oral cavity comprises different anatomical sub-sites: buccal mucosa, anterior two thirds of the mobile tongue (oral tongue), upper and lower alveolar ridge, retromolar trigone (retromolar gingiva), floor of the mouth and hard palate [3]. These anatomical sites have a rich lymphatic drainage and regional lymph node metastasis are typically predictable and vary according to the primary site. For instance, hard palate cancer has a metastatic potential to buccinator, submandibular and jugular nodes; whereas primary anterior oral tongue cancer mainly spread to submandibular, upper- and middle-jugular nodes (bilaterally when the lesion is close to the midline) [3]. Similarly, the estimated risk of lymph node involvement at diagnosis, accounting for nearly 30% of cases, is strictly related to primary sub-site. Primaries of the anterior tongue frequently (50–60%) involve the neck, whereas lymph node metastasis are infrequently in patients with alveolar ridge and hard palate cancers [3]. Consequently, based on the selected primary end-point, it would be more relevant to select adequate primary sub-site to increase study power and obtain a high-quality result. Moreover, the choice of the best comparator is an important question. It should be highlighted that the American joint committee on cancer (AJCC) staging manual introduces significant modifications in the oral cavity section [76]. The main changes include the update to the tumor (T) category, including the depth of invasion (T1: tumors ≤ 2 cm and with ≤ 5 mm depth of invasion; T2: tumors ≤ 2 cm and > 5 mm but ≤ 10 mm depth of invasion or tumor > 2 cm but ≤ 4 cm and ≤ 10 mm depth of invasion; T3: tumor > 4 cm or any tumor > 10 mm depth of invasion) and the addition of extracapsular extension to nodal (N) category (N2a: Metastasis in a single ipsilateral or contralateral lymph node ≤ 3 cm in greatest dimension and with extracapsular extension or metastasis in a single ipsilateral lymph node > 3 cm but ≤ 6 cm in greatest dimension and extracapsular extension negative; N3b: Metastasis in a single ipsilateral node more than 3 cm in greatest dimension and extracapsular extension positive; or metastasis in multiple ipsilateral, contralateral, or bilateral lymph nodes, with any extracapsular extension positive) [76]. These modifications make directly comparison with matched control group or published data more difficult. It is plausible that in the future a methodologically robust study design would be the ideal solution to improve our knowledge about the nanotechnology effectiveness in OCC. 

For sure, a number of challenges must be overcome before nanotechnology can be introduced into routine OCC clinical practice. However, its potential applicability needs to be highlighted. Nanotechnology could represent a solution (i) for the definition of an accurate imaging protocol to stage disease at diagnosis; (ii) for the early lymph nodes evaluation during surgery and (iii) for the management of a second primary or recurrent OCC in a previously treated area—repeat surgery is often difficult due to progressive tissue loss and repeat RT is often difficult due to dose constraints of organs at risk. Therefore, the field of nanotechnology seems to be promising for improving both life expectancy and quality of the OCC patients. It is clear that prospective studies are needed to define treatment strategies.

## Figures and Tables

**Table 1:** Nanocarriers for oral cavity carcinoma.

Nanomaterial	Structure	Advantages	Limitations
Nanoparticle	polysaccharides, proteins and biocompatible/biodegradable polymers	biocompatibility, biodegradability	toxicity?
Liposome	membrane-like lipid layers, often phospholipids and cholesterol	permeability, charge density, steric hindrance	Less stable than nanoparticles
Hydrogel	hydrophilic polymeric chains dispersed in water	hydrophilicity, flexibility, versatility, high water absorptivity, biocompatibility; it interacts with saliva glycoproteins, causing mucoadhesion	slow response time
Liquid crystal	materials in mesophase state	it can be stored for long periods because thermodynamically stable	toxicity?

PI3K/Akt/mTOR: phosphatidylinositol 3-kinase/Akt/mammalian target of rapamycin.

**Table 2:** Patterns of lymph node enhancement for ferumoxtran 10-enhanced magnetic resonance imaging [27].

Diagnosis	Patterns Post Contrast	Characteristic
Non-pathologic node	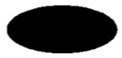	Node having an overall dark signal intensity; homogenous architecture
Non-pathologic node	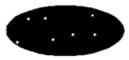	Node having an overall dark signal with subtle granularities; homogenous architecture
Non-pathologic node	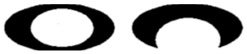	Node having an overall dark signal other than a central or hilar area of fat seen on T1 sequence; heterogenous architecture
Possibly pathologic node	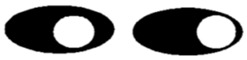	Less than 50% of node has high signal intensity; heterogenous architecture
Pathologic node	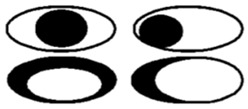	Partial darkening whereby more than 50% of the node has area of high signal intensity; heterogenous architecture
Pathologic node	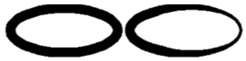	Node has central high signal with darkening along the peripheral rim; heterogenous architecture
Pathologic node	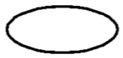	No blackening of node or node hyperintense to surrounding tissue; is heterogenous or homogenous architecture

**Table 3:** Photodynamic therapy in the treatment of oral cavity carcinoma.

Author	Year	Study Type	OCC Patients	Photosensitizers	Outcomes
Karakullukcu [70]	2011	Retrospective	105	Temoporfin	OR: 91.4%; CR: 68.6%; 2-y DFS: 74%; 5-yDFS: 61%
Biel [71]	2007	Retrospective	161	Photofrin	CR: 93.2%
Schweitzer [72]	2001	Retrospective	10	Photofrin	CR: 80%
Grant [73]	1993	Retrospective	11	Photofrin	CR: 90.9%
Hopper [74]	2004	Phase IIb	121	Meta-tetrahydroxyphenylchlorin	CR: 85%; 1-y OS: 89%; 2-y OS: 75%

OCC: oral cavity carcinoma; OR: overall response; CR: complete response; 2-y DFS: 2-year disease-free survival; 5-y DFS: 5-year disease-free survival; 1-y OS: 1-year overall survival; 2-y OS: 2-year overall survival.
